# Differential expression of eicosanoid pathways after whole blood stimulation in asthma patients^☆^

**DOI:** 10.1016/j.waojou.2025.101047

**Published:** 2025-04-02

**Authors:** Chrysanthi Skevaki, Pavel Tafo, Thomas Bahmer, Mustafa Abdo, Henrik Watz, Frauke Pedersen, Christian Herzmann, Klaus F. Rabe, Harald Renz, Wolfgang Andreas Nockher, the ALLIANCE Study Group as part of the German Center for Lung Research (DZL), Mira Bürk, Markus Ege, Alexander Hose, Sabina Illi, Constanze Jakwerth, Kristina Laubhahn, Lena Lagally, Nicole Maison, Jimmy Omony, Bianca Schaub, Carsten Schmidt-Weber, Lena Ullemeyer, Erika von Mutius, Esther Zeitlmann, Ulrich Zissler, Mustafa Abdo, Thomas Bahmer, Heike Biller, Xenia Bovermann, Folke Brinkmann, Karoline I. Gaede, Christian Herzmann, Clara Haug, Berrit Liselotte Husstedt, Nikolas Jacobs, Anne-Marie Kirsten, Inke R. König, Matthias V. Kopp, Gyde Nissen, Catharina Nitsche, Frauke Pedersen, Klaus F. Rabe, Isabell Ricklefs, Alena Steinmetz, Lea Kronziel, Vera Veith, Gesche Voigt, Henrik Watz, Markus Weckmann, Mifflin-Rae Calvero, David S. DeLuca, Anna-Maria Dittrich, Christian Dopfer, Svenja Gaedcke, Ruth Grychtol, Anika Habener, Gesine Hansen, Christine Happle, Adan Chari Jirmo, Bin Liu, Lennart Riemann, Nicolaus Schwerk, Svenja Foth, Harald Renz, Christin Langer, Stefanie Weber, Miguel A. Alejandre Alcazar, Samira Blau, Silke van Koningsbruggen-Rietschel, Ernst Rietschel, Tobias Trojan

**Affiliations:** f Department of Paediatric Allergology, Dr von Hauner Children's Hospital, Ludwig Maximilians University, Munich, Germany; g Clinical Respiratory Epidemiology, Dr. von Hauner Children's Hospital, Ludwig Maximilian University of Munich, Munich, Germany; h Institut für Asthma- und Allergieprävention (IAP), Helmholtz Zentrum Munich, Deutsches Forschungszentrum für Gesundheit und Umwelt (GmbH), Munich, Germany; i Center of Allergy & Environment (ZAUM), Technical University of Munich and Helmholtz Center Munich, German Research Center for Environmental Health, Munich, Germany; j LungenClinic Grosshansdorf GmbH, Grosshansdorf, Germany; k University Children's Hospital, Luebeck, Germany; l Research Center Borstel – Medical Clinic, Borstel, Germany; m Pulmonary Research Institute at LungenClinic Grosshansdorf, Grosshansdorf, Germany; n Institute for Medical Biometry and Statistics, University Luebeck, University Medical, Centre Schleswig-Holstein, Campus Luebeck, Germany; o Department of Paediatric Respiratory Medicine, Inselspital, University Children's Hospital of Bern, University of Bern, Bern, Switzerland; p Hannover Medical School, Hannover, Germany; q Department of Paediatric Pneumology, Allergology and Neonatology, Hannover Medical School, Hannover, Germany; r University Children's Hospital Marburg, University of Marburg, Germany; s Institute of Laboratory Medicine and Pathobiochemistry, Molecular Diagnostics, University of Marburg, Germany; t University of Cologne, Faculty of Medicine and University Hospital Cologne, Translational Experimental Pediatrics - Experimental Pulmonology, Department of Pediatric and Adolescent Medicine, Germany; u Excellence Cluster on Stress Responses in Aging-associated Diseases (CECAD), University of Cologne, Faculty of Medicine and University Hospital Cologne Cologne, Germany; v Institute for Lung Health, University of Giessen and Marburg Lung Centre (UGMLC), Member of the German Centre for Lung Research (DZL), Gießen, Germany; w University of Cologne, Faculty of Medicine and University Hospital Cologne, Department of Pediatrics, Cologne, Germany; x Comprehensive Pneumology Center, Munich (CPC-M), Germany; y German Center for Lung Research (DZL), Germany; z German Center for Lung Research (DZL), Munich, Germany; aa Airway Research Center North (ARCN), Germany; ab Biomedical Research in Endstage and Obstructive Lung Disease Hannover (BREATH), Germany; ac University of Giessen Marburg Lung Center (UGMLC), Germany; ad Member of the German Center for Lung Research, Germany; ae University of Gießen, Marburg Lung Center (UGMLC), Germany; a Institute of Laboratory Medicine and Pathobiochemistry, Molecular Diagnostics, Philipps-University Marburg, University of Giessen Marburg Lung Center (UGMLC), German Center for Lung Research (DZL), Marburg, Germany; b LungenClinic Grosshansdorf GmbH, Airway Research Center North (ARCN), German Center for Lung Research (DZL), Grosshansdorf, Germany; c Universitätsklinikum Schleswig-Holstein Campus Kiel, Internal Medicine Department I, Airway Research Center North (ARCN), German Center for Lung Research (DZL), Kiel, Germany; d Pulmonary Research Institute at LungenClinic Grosshansdorf GmbH, Airway Research Center North (ARCN), German Center for Lung Research (DZL), Grosshansdorf, Germany; e Forschungszentrum Borstel, Klinisches Studienzentrum, Airway Research Center North (ARCN), German Center for Lung Research (DZL), Borstel, Germany

**Keywords:** Eicosanoids, Asthma, Inflammation

## Abstract

**Objectives:**

Asthma is a heterogeneous disease regarding its pathophysiology, clinical symptoms, and response to treatment. Eicosanoids are important inflammatory mediators, able to either promote or attenuate the underlying chronic airway inflammation. We compared eicosanoid expression patterns in the blood circulation and in stimulated blood leukocytes of asthma patients to identify differences in eicosanoid release which may be related to airway inflammation.

**Methods:**

Blood was collected from 198 adult asthmatic patients and 63 healthy controls, participating in the German Center for Lung Research (DZL) ALLIANCE cohort. Eicosanoid release from leukocytes was analyzed using heparinized whole blood after *in vitro* stimulation with zymosan. Additionally, circulating eicosanoids were measured directly from ethylenediaminetetraacetic acid (EDTA) plasma. Eicosanoids were extracted via solid phase extraction and quantified by high-performance-liquid-chromatography-tandem-mass-spectrometry (HPLC-MS^2^).

**Results:**

Eicosanoid levels were low in blood circulation with no significant differences between asthmatics and controls, except for leukotriene E_4_ (LTE_4_) which was slightly elevated in asthmatics. After *in vitro* stimulation we observed an inhibition of prostaglandin and thromboxane biosynthesis only in patients with severe asthma which was related to the regular use of systemic corticosteroids. In contrast, a significant increase was shown for formation of the 5-Lipoxygenase (5-LOX) product LTE_4_ in steroid-naïve asthmatics with moderate as well as severe disease severity but not in subjects with systemic steroid treatment. Furthermore 15-Hydorxyeicosatetraenoic acid (15-HETE) production was elevated in asthmatic patients with mild-to-moderate disease activity but dropped down in severe asthmatics.

**Conclusions:**

Profiling of eicosanoid production in stimulated whole blood samples showed a specific biosynthesis pattern of asthmatic patients, which is influenced by the use of systemic corticosteroids.

## Introduction

Asthma is a multifactorial disorder initiated by both genetic and environmental factors and is characterized by heterogeneity in clinical presentation and the type and intensity of airway inflammation and remodeling.^1^ Despite this clinical and molecular variability, searching for biomarkers is important to measure disease activity to develop risk stratifications or treatment identification and monitoring clinical response.^2^ For asthma phenotyping, the majority of studies focused on the investigation of chemokines, cytokines as well as their cellular receptors, as they are considered to be the most prominent mediators of chronic airway inflammation.^3^ However, also eicosanoids are involved in several pathophysiological processes relevant to asthma or other chronic inflammatory diseases.^4^

Eicosanoids are biologically active lipid mediators, synthesized by enzymatic metabolization of arachidonic or other polyunsaturated fatty acids liberated from cellular membrane phospholipids.^5^ Depending on activation of the particular enzymatic pathways, the biological active compounds released are prostaglandins, leukotrienes, thromboxanes, or lipoxins. Within an ongoing inflammatory process, eicosanoid signaling can result in enhancement as well as silencing of the inflammation depending on the nature of these mediators.^6^ Eicosanoids are mainly produced by activated blood phagocytes, such as eosinophilic or neutrophilic granulocytes, and monocytes/macrophages,^7^ and these cells are important components of the inflammatory tissue infiltrate in the allergic airways.^8^^,^^9^ Leukotriene 4 (LTE 4) is an important player in airway inflammation, predominantly acting through the CysLT1, CysLT2, and the CysLT3 (OPR99) receptors and causing epithelial swelling and increased vascular permeability.^10^ Along these lines, high levels of LTE4 and prostaglandin 2 (PGD2) metabolites are frequently observed in patients with asthma.^11^ Higher concentrations of these metabolites were linked to reduced lung function, increased exhaled nitric oxide, and eosinophil markers in blood, sputum, and urine among patients with asthma.^12^

The All Age Asthma Cohort (ALLIANCE) of the German Center for Lung Research (DZL)^13^ offers an inter-disciplinary framework to study disease mechanisms and to identify biomarkers and predictors for distinct asthma trajectories. In the present study we hypothesized that asthma patients show an altered eicosanoid expression pattern after zymosan stimulation of whole blood samples. This fungal derived polysaccharide specifically activates leukocytes and macrophages via activation of the TLR2 receptor and results in arachidonic acid mobilization^14^^,^^15^ and production of inflammatory mediators.^16^^,^^17^

## Materials and methods

### Study population

The ALLIANCE cohort of the DZL is a prospective, multi-center, observational cohort study with 7 recruiting sites across Germany.^13^ Patients in the adult arm of the ALLIANCE cohort (ClinicalTrials.Gov: NCT02419274) were recruited at LungenClinic Grosshansdorf and at the Medical Clinical Research Center Borstel between March 2014 and May 2018. Detailed information on recruitment, including in-, and exclusion criteria has been described previously.^13^ The current analysis includes adult subjects (aged 19–81 years) with either mild-to-moderate asthma (N = 108) or severe asthma (N = 90) and healthy controls (N = 63). Asthma severity was assessed according to the European Respiratory Society (ERS)/American Thoracic Society (ATS) guidelines 2014, including lung function, exacerbation frequency, asthma control, and medication [Supplemental References SR1]. Essential subject characteristics are summarized in Table 1.

**Table 1:** **Demographic and clinical characteristics of subjects included in this study.** Unless otherwise stated, data represents mean values ± SD

	Healthy controls	Patients with asthma	p-value
Subjects	63	198	
Age (years)	50.3 ± 17.5	51.9 ± 13.8	0.509
Sex			0.188
Male (n,%)	34 (54.0%)	86 (43.4%)	
Female (n,%)	29 (46.0%)	112 (56.6%)	
BMI	24.7 ± 3.59	27.6 ± 5.24	<0.001
Smoking status (n,%)			0.634
Never or former smoker (<10PY)	49 (77.8%)	146 (73.7%)	
Current or former smoker (≥10 PY)	14 (22.2%)	52 (26.3%)	
White blood cells [10^6^/ml]	6.02 ± 1.46	7.87 ± 2.61	<0.001
Blood eosinophiles [%]	2.91 ± 1.67	4.66 ± 3.62	<0.001
Asthma severity (n)			
Mild-to-moderate	–	108	
Severe	–	90	
FEV_1_ (pre SABA) [%pred.]	103 ± 10.6	80.3 ± 22.1	<0.001
FeNO [ppb]	17.9 ± 9.77	38.8 ± 42.3	<0.001
Exacerbations^a^	–	1.14 ± 1.09	
ICS dose (fluticasone-equivalent) [μg/day]	–	538.2 ± 479.5	
Subjects under OCS therapy (n)			
Mild-to-moderate	–	–	
Severe	–	44	

a Number of severe exacerbations within the previous 12 months requiring a steroid burst of at least 3 days OCS treatment.

The study was approved by the local ethics committee of the University of Lübeck, Germany (Az.12-215). All patients provided written informed consent prior to inclusion in this study.

### Chemicals and standards

Eicosanoid standards and deuterated internal standards were purchased from Cayman Chemical (local distributor: Biomol, Hamburg, Germany). Liquid chromatography-mass spectrometry (LCMS) grade solvents were purchased from VWR, Merck (both Darmstadt, Germany), Honeywell (Seelze, Germany) and Fisher Scientific (Schwerte, Germany). RPMI Medium and zymosan A from *Saccharomyces cerevisiae* were obtained from Sigma-Aldrich (St. Louis, MO, USA). l-glutamine was purchased from PAA Laboratories GmbH (Pasching, Austria).

### Blood sampling and stimulation procedure

Venous blood samples were collected by venipuncture in lithium-heparin monovettes and ethylenediaminetetraacetic acid (EDTA) monovettes (Sarstedt, Nümbrecht, Germany). Within 1 h after blood donation, 2 ml heparinized blood were mixed with 1 ml RPMI medium containing 4 mM Lglutamine and 750 μg/ml zymosan (final concentration 250 μg/ml). Samples were incubated at 37 °C for 4 h. Afterwards, samples were centrifuged at 1000 g at room temperature for 5 min. After separation of cell pellet and plasma using a seraplas filter (Sarstedt, Nümbrecht, Germany), 1 ml of the supernatant was transferred into a 2 ml reaction tube and stored at −80 °C until further processing. For analysis of baseline concentrations of lipid mediators, EDTA plasma was generated by centrifugation as described above and 0.5 ml of the supernatant was stored at 80 °C until further processing.

### Eicosanoid extraction

Eicosanoids were extracted as described previously [SR2, SR3]. Briefly, 25 μl of the deuterated internal standard mixture (Supplemental Table 1) and 50 μl methanol were added to 1 ml of the supernatants and mixed vigorously. After centrifugation (10,000 rpm, 4 °C, 5 min) eicosanoids were extracted using Bond Elute Plexa solid phase extraction columns (Agilent Technologies, Santa Clara, CA, USA), following the manufacturer's instructions. Analytes were eluted using 500 μl methanol. Extracts were evaporated, resuspended in 100 μl of water/acetonitrile/formic acid (70:30:0.02, v/v/v; solvent A) and subsequently analyzed by HPLC-MS^2^.

### Mass spectrometry and data analysis

Eicosanoids were analyzed using an Agilent 1290 infinity LC system (Agilent Technologies, Santa Clara, CA, USA) coupled to an electrospray interface of a QTRAP 5500 mass spectrometer (Sciex, Darmstadt, Germany). Samples were separated on a Synergi Hydro reverse-phase C18 column (2.1 × 250 mm; Phenomenex, Aschaffenburg, Germany) with a flow rate of 0.3 ml/min using the same gradient as described previously [SR3]. The column was re-equilibrated by keeping 0% solvent B (acetonitrile/isopropyl alcohol, 50:50, v/v) for 5 min. Compounds were detected in negative scheduled multiple reaction monitoring mode, using Q1/Q3 transitions, individual collision energies and declustering potentials as described previously [SR2]. An external 10-point calibration curve was used for quantification. Data analysis and integration of peaks was performed using the MultiQuant software (v.2.1.1, Sciex, Darmstadt, Germany). A detailed overview of analyzed compounds is given in Supplemental Table 2.

### Statistical analyses

Data processing and statistical analysis was performed with R (version 4.0.2) [SR4]. Prior to statistical analysis, data from the non-stimulated plasma samples were subtracted from the values of the stimulation, to correct for basal eicosanoid levels. Furthermore, data were normalized to the total white blood cell count, to eliminate the effect of differential leukocyte count between asthmatics and healthy controls. For statistical analysis, metabolites were excluded, which were below the detection limit in at least 80% of all samples in both groups. Remaining left-censored values below the limit of detection were replaced by zero. A sensitivity analysis shows no difference between this approach and various other approaches such as substitution with $\frac{LOD}{\sqrt{2}}$ or Kaplan-Meier estimation [SR5-SR7].

Pairwise comparisons were performed using the Wilcoxon rank-sum test [SR8] and p values were assessed through permutation (10,000 permutations of the group labels). Pvalues were adjusted by the step-down algorithm to control the false discovery rate [SR9].

Pathway analysis was done by functional class scoring (FCS) which includes either building a pathway-level statistic based on all metabolite-level statistics or directly computing a multivariate pathway-level statistic [SR10]. The obtained p-values from the pairwise comparisons were used as metabolite-level statistics and then combined within each pathway to a pathway-level statistic. The p-values were combined using the Tippet combining function [SR11] and adjusted with the step-down algorithm for control of the false discovery rate.

## Results

### Low baseline concentrations of eicosanoid mediators in human plasma samples

To determine the optimal pre-analytical conditions of eicosanoid analysis in human blood, we first compared eicosanoid levels obtained from specimens collected in serum coagulation tubes or EDTA- or Li-heparin anti-coagulation tubes up to 4 h after venipuncture. Especially metabolites of the cyclooxygenase (COX) as well as of the 12- and 15-LOX pathway were higher in serum compared to heparin and even less in EDTA plasma tubes over time (data not shown). Therefore, we assumed that eicosanoid concentrations in EDTA plasma were more reliable to indicate endogenous eicosanoid levels in the blood circulation.

In total, we could detect 41 out of 67 different eicosanoids in human plasma samples from asthmatic patients and healthy controls (Table 2). However, the average concentration of most eicosanoids was very low and often close to the limit of detection. Only the 2 hydroxyoctadecadienoic acids (9-/13-HODE) and 2 metabolites of the cytochrome P450 monooxygenase (CYP450) pathway were found at higher concentrations. Comparison of plasma eicosanoid concentrations between asthmatic subjects and healthy controls did not show significant differences at baseline levels, except for LTE_4_ which was slightly elevated in asthmatics.

**Table 2:** **Baseline eicosanoid levels, in human plasma.** Metabolites are abbreviated according to the common nomenclature for eicosanoids [SR12, SR13]. Values represent Mean ± Standard deviation

Metabolite	Pathway	Mean concentration [ng/ml]		p-value
		Healthy controls (n = 63)	Asthma (n = 198)	
12-HETE	12-LOX	0.503 ± 0.524	0.458 ± 0.653	1.000
Tetranor-12-HETE	12-LOX	0.029 ± 0.014	0.033 ± 0.021	1.000
12-oxoETE	12-LOX	ND^a^	ND	
14-HDoHE	12-LOX	0.151 ± 0.207	0.146 ± 0.282	1.000
Maresin 1	12-LOX	ND	ND	
12-HEPE	12-LOX	0.108 ± 0.154	0.092 ± 0.181	0.994
15-HETE	15-LOX	0.277 ± 0.162	0.298 ± 0.252	1.000
15-oxoETE	15-LOX	0.005 ± 0.012	0.026 ± 0.059	0.714
8-HETE	15-LOX	0.045 ± 0.031	0.054 ± 0.035	0.999
14,15-LTC_4_ (Eoxin C_4_)	15-LOX	ND	ND	
8,15-diHETE	15-LOX	ND	ND	
LXA_4_	15-LOX	ND	ND	
17-HDoHE	15-LOX	0.214 ± 0.164	0.217 ± 0.325	0.998
10,17-DiHoHE (NPD_1_)	15-LOX	ND	ND	
7,17-dihydroxy-DPA	15-LOX	ND	ND	
15-HEPE	15-LOX	0.032 ± 0.030	0.045 ± 0.053	0.993
13-HODE	15-LOX	4.394 ± 4.625	4.331 ± 3.978	1.000
5-HETE	5-LOX	0.141 ± 0.058	0.143 ± 0.080	1.000
5-oxoETE	5-LOX	0.011 ± 0.010	0.013 ± 0.011	0.986
LTE_4_	5-LOX	0.001 ± 0.001	0.002 ± 0.003	**0.028**
20-carboxy-LTB_4_	5-LOX	ND	ND	
20-hydroxy-LTB_4_	5-LOX	ND	ND	
LTB_4_	5-LOX	ND	ND	
LTC_4_	5-LOX	ND	ND	
LTD_4_	5-LOX	ND	ND	
5,15-diHETE	5-LOX/15-LOX	ND	ND	
4-HDoHE	5-LOX	0.102 ± 0.104	0.073 ± 0.073	1.000
7-HDoHE	5-LOX	0.015 ± 0.018	0.021 ± 0.025	0.992
RvD_2_	5-LOX/15-LOX	ND	ND	
5-HEPE	5-LOX	0.032 ± 0.023	0.037 ± 0.028	0.999
RvE_1_	5-LOX	ND	ND	
11-HETE	COX	0.042 ± 0.039	0.030 ± 0.024	1.000
12-HHT	COX	0.259 ± 0.348	0.247 ± 0.423	1.000
6-keto-PGF_1α_	COX	0.014 ± 0.013	0.019 ± 0.011	1.000

PGA_2_	COX	0.005 ± 0.005	0.006 ± 0.006	0.999
PGE_2_	COX	0.082 ± 0.402	0.091 ± 0.512	0.376
PGF_2α_	COX	0.032 ± 0.013	0.036 ± 0.013	0.992
Tetranor-PGEM	COX	0.075 ± 0.102	0.048 ± 0.094	0.306
TXB_2_	COX	0.096 ± 0.177	0.122 ± 0.238	1.000
11-dhTXB_2_	COX	ND	ND	
15-deoxy-PGJ_2_	COX	ND	ND	
6,15-diketo-13,14-dihydro-PGF1_α_	COX	ND	ND	
PGD_2_	COX	ND	ND	
PGF_1α_	COX	0.013 ± 0.011	0.017 ± 0.010	1.000
17-keto-DPA	COX	0.021 ± 0.043	0.014 ± 0.035	0.930
PGF_3α_	COX	0.008 ± 0.010	0.009 ± 0.010	1.000
11-HEPE	COX	ND	ND	
PGD_3_	COX	ND	ND	
PGE_3_	COX	ND	ND	
TXB_3_	COX	ND	ND	
9-HODE	COX	2.179 ± 1.822	2.273 ± 1.991	1.000
9-HpODE	COX	ND	ND	
11,12-DHET	CYP	0.283 ± 0.121	0.252 ± 0.119	0.228
11,12-EET	CYP	0.003 ± 0.004	0.003 ± 0.003	0.999
14,15-DHET	CYP	0.351 ± 0.285	0.354 ± 0.307	0.999
18-HETE	CYP	0.148 ± 0.116	0.177 ± 0.144	1.000
20-HETE	CYP	ND	ND	
14,15-diHETE	CYP	0.103 ± 0.047	0.109 ± 0.060	0.998
12,13-diHOME	CYP	4.328 ± 4.238	3.162 ± 2.838	0.545
12,13-EpOME	CYP	0.414 ± 0.478	0.550 ± 0.653	0.996
9,10-diHOME	CYP	1.548 ± 2.013	1.235 ± 1.297	1.000
18-HEPE	CYP/COX	0.027 ± 0.030	0.024 ± 0.026	1.000
5-iso-PGF_2α_	Auto oxidation	0.051 ± 0.021	0.140 ± 0.840	0.986
9-HETE	Auto oxidation	0.031 ± 0.018	0.027 ± 0.020	0.939
10-HDoHE	Auto oxidation	0.029 ± 0.036	0.018 ± 0.020	1.000
9-HEPE	Auto oxidation	0.004 ± 0.009	0.003 ± 0.013	0.297
8-HEPE	Auto oxidation	ND	ND	

a ND: not detected.

### Differential expression of eicosanoid pathways in asthmatic patients in whole blood stimulations

Next, we investigated changes in eicosanoid biosynthesis in asthmatic subjects and healthy controls after *in vitro* whole blood stimulation with zymosan for 4 h. The asthmatic population was further divided into severe and mild-to moderate asthmatics depending on clinical parameters.

**Fig. 1:**
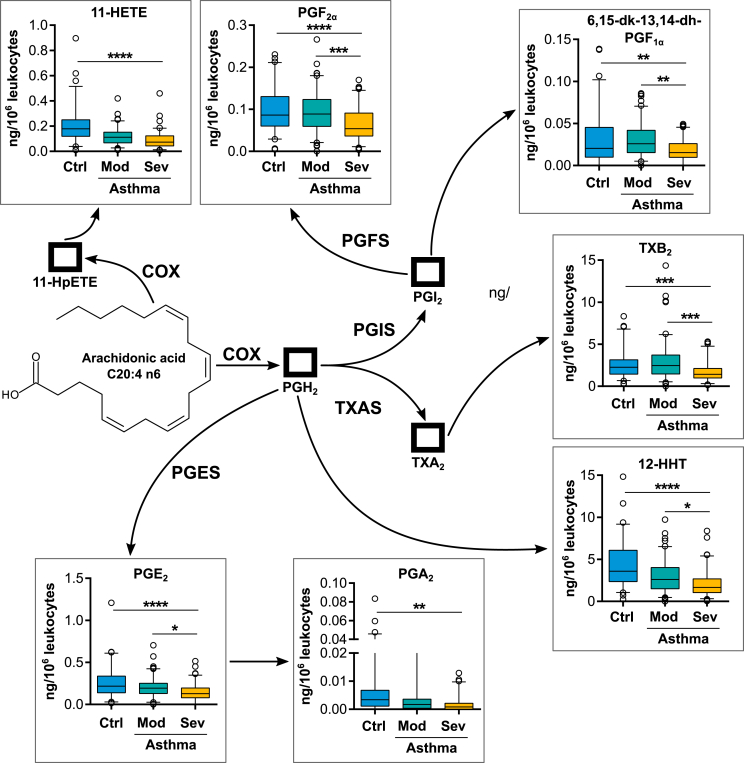
**Expression of COX derived eicosanoids after whole blood stimulation with zymosan.** Metabolite levels of healthy controls (Ctrl) were compared to asthmatic subjects which were grouped according to their asthma severity into mild-to-moderate (Mod) and severe (Sev) asthmatics. ∗p-value ≤0.05, ∗∗ p-value ≤0.01, ∗∗∗p-value ≤0.001, ∗∗∗∗p-value ≤0.0001. Abbreviations: COX: cyclooxygenase, PGES: prostaglandin E synthase, PGFS: prostaglandin F synthase, PGIS: prostaglandin I synthase, TXAS: thromboxane A synthase

In total we detected 51 different eicosanoids, belonging to all 5 main eicosanoid biosynthesis pathways. In order to identify differentially regulated eicosanoid biosynthesis pathways between asthmatic subjects and healthy controls, we first performed a whole pathway analysis using a functional class scoring approach [SR10]. Therefore, metabolites were grouped according to their assignment to the different pathways. Metabolites that are produced predominantly via auto-oxidation processes were not included. The pathway analysis revealed a differential expression of all main eicosanoid pathways between the asthmatic population and healthy controls (Table 3, Supplemental Fig. 1). However, this analysis does not provide information about the direction of the changes. Therefore, we directly compared metabolite levels between healthy controls and asthmatic patients with different disease severity. In patients with severe disease activity, we found a decreased production of metabolites of the COX-pathway, ie, PGE_2_ and its dehydration product PGA_2,_ 11HETE, 12-HHT, TXB_2_ as well as PGF_2α_, compared to healthy controls (Fig. 1). Moreover, the concentration of several eicosanoids of this pathway inversely correlated with disease severity, as they were significantly decreased in severe asthmatics compared to the mild-to-moderate group (Fig. 1).

**Table 3:** **Pathway analysis to identify differentially expressed eicosanoid biosynthesis pathways in asthmatic patients.** Number of metabolites included for each pathway are indicated

Pathway	Metabolites included	p-value
**COX**	10	0.0001
**5-LOX**	8	0.0001
**15-LOX**	4	0.0003
**CYP**	5	0.0001

**Fig. 2:**
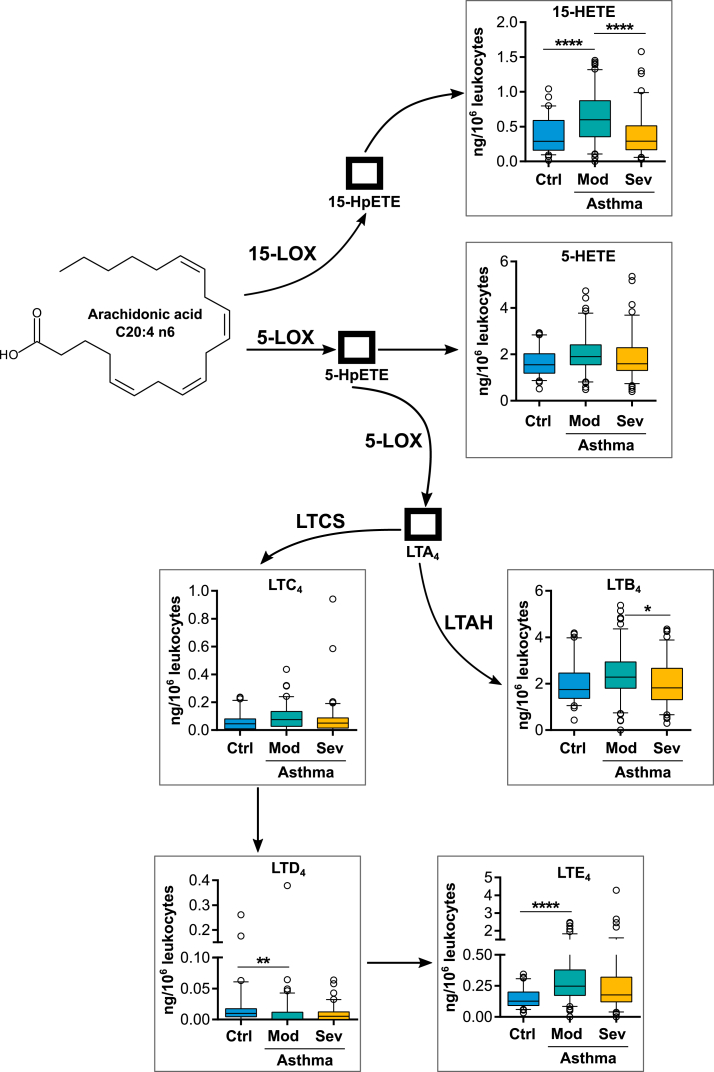
**Expression of LOX derived eicosanoids after whole blood stimulation with zymosan.** Metabolite levels of healthy controls (Ctrl) were compared to asthmatic subjects which were grouped according to their asthma severity into mild-to-moderate (Mod) and severe (Sev) asthmatics. ∗p-value ≤0.05, ∗∗ p-value ≤0.01, ∗∗∗p-value ≤0.001, ∗∗∗∗p-value ≤0.0001. Abbreviations: 5(15)-LOX: 5(15)-lipoxygenase, LTCS: leukotriene C_4_ synthase, LTAH: leukotriene A_4_ hydrolase

Activity of the 5-LOX pathway, resulted in increased production of LTE_4_ in asthmatic patients while its precursor LTD_4_ showed slightly decreased levels compared to healthy controls, but the concentrations of this intermediate were extremely low (Fig. 2). Interestingly, LTE_4_ expression was mainly elevated in mild-to-moderate asthmatics while its concentration decreased in patients with severe disease. Among the other metabolites of the 5-LOX pathway, only LTB_4_ showed slightly decreased concentrations in severe asthmatics compared to patients with mild-to-moderate disease (Fig. 2).

Investigating eicosanoids from the 15-LOX pathway we found the main metabolite 15-HETE to be elevated with mild-to-moderate disease activity compared to healthy controls (Fig. 2). Interestingly, this metabolite concentration again inversely correlated with disease severity, as production of 15-HETE in severe asthmatics was significantly decreased compared to the mild-to moderate group.

Finally, we only observed minor changes for a few eicosanoids belonging to other LOX as well as the CYP450 monooxygenase pathway (Supplemental Fig. 2).

### Effects of oral glucocorticoids on stimulated eicosanoid pathway expression

Oral corticosteroids (OCS) are used for the treatment of severe asthma when application of local inhaled steroids is insufficient to control disease activity. As OCS show profound anti-inflammatory activity, we compared eicosanoid expression in severe asthmatics with (n = 44) and without (n = 46) permanent use of OCS. Patients in the mild-to-moderate group did not use OCS.

**Fig. 3:**
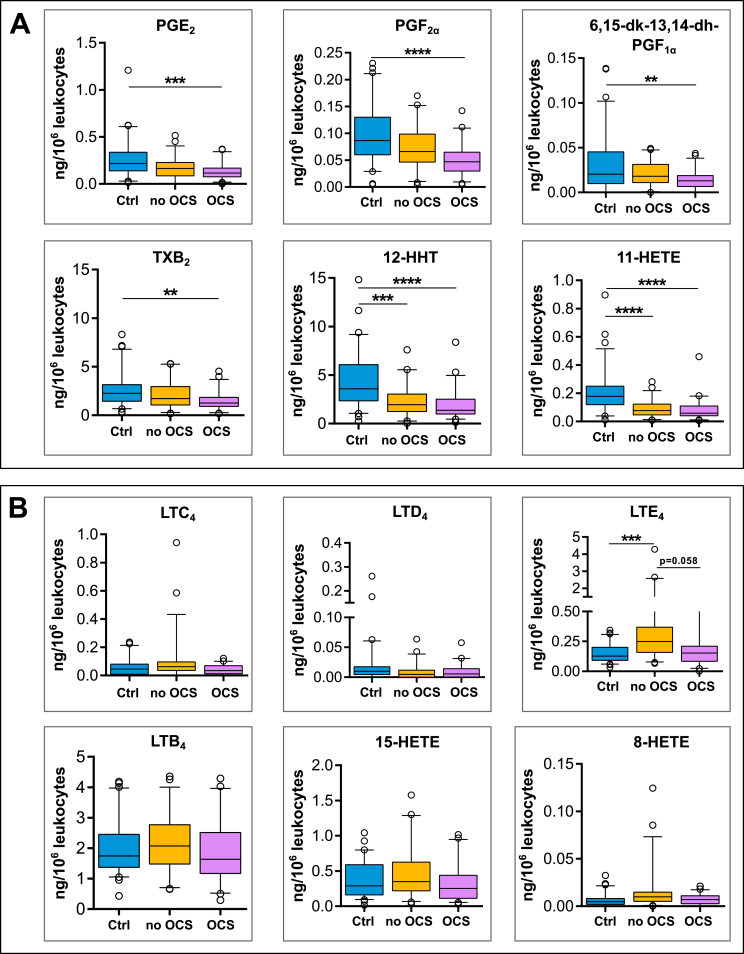
**Effects of oral glucocorticoids (OCS) on expression of COX (A) and LOX (B) derived eicosanoids in zymosan stimulated whole blood.** Metabolite levels of healthy controls (Ctrl) were compared to subjects with severe asthma grouped into steroid naïve (no OCS) and steroid treated (OCS) asthmatics. ∗p-value ≤0.05, ∗∗ p-value ≤0.01, ∗∗∗p-value ≤0.001, ∗∗∗∗p-value ≤0.0001

Patients with permanent OCS-intake showed the lowest expression of COX pathway metabolites, which all were significantly decreased compared to healthy controls (Fig. 3A). In contrast, severe asthmatics without OCS treatment only showed decreased metabolite levels for 12-HHT and 11-HETE. The levels of main prostanoids such as PGE_2_ and thromboxane were also lower in this group compared to healthy controls, but this does not reach statistical significance. Therefore, the observed suppression of COX-derived eicosanoids in asthmatic patients is mainly influenced by OCS therapy (Fig. 3 A).

Furthermore, the formation of LTE_4_ from the 5-LOX pathway was found to be lower in asthmatics with OCS even though this suppression was not statistically significant (Fig. 3B). However, LTE_4_ synthesis in patients with severe disease activity and without OCS treatment was elevated compared to healthy controls such as found for the mild-to-moderate patient group without any OCS application. Finally, OCS therapy had no effect on LTB_4_ and 15-HETE production.

## Discussion

Asthma is a heterogenous disease, characterized by airway hyperresponsiveness, variable airway obstruction and chronic airway inflammation. Almost all cell types involved in inflammatory processes are able to produce eicosanoids or express their corresponding receptors on the cell surface.^18^ Leukotrienes are the most potent bronchoconstrictors in humans,^4^^,^^19^ potentiate airway hyperresponsiveness to allergens^20^^,^^21^ and are increased in asthmatic subjects during exacerbations.^22^ In contrast, cyclooxygenase derived prostaglandins have pleiotropic effects on the pathophysiology of asthma. Whereas PGD_2_ promotes type 2 inflammation by recruiting Th2 cells, eosinophils and basophiles^23^ as well as by inducing IL-4, IL-5 and IL-13 production,^24^ PGE_2_ and PGI_2_ have shown to inhibit cytokine release from Th2 CD4 T cells and macrophages.^25^^,^^26^

After analyzing basal eicosanoid levels from EDTA plasma only a limited number of compounds could be detected, most of them at low concentrations close to the limit of detection. Further, the only significant difference between asthmatic subjects and healthy controls were found for LTE_4_. However, average concentrations of this metabolite in plasma are very low in both groups, thus a biological effect is very unlikely, as binding affinities of LTE_4_ for the human CysLT-receptors were shown to be in the nanomolar range.^27^^,^^28^ As eicosanoids are normally only synthesized on demand and characterized by short half-lives and rapid local degradation, the overall low concentrations found in the circulation of study participants seems reasonable. Yasumoto et al made similar observations, only detecting a few eicosanoids from COX and LOX pathways but several intermediates of the CYP pathway in citrate plasma of healthy volunteers.^29^ In contrast, Zhou et al found significant differences in plasma eicosanoid levels in a small cohort of asthmatic patients compared to healthy controls. Especially PGE_2_, TXB_2_ and PGE_1_ were elevated in asthmatic patients.^30^ However, the overall differences between the groups were rather small. Further, the authors did not provide information about inclusion or exclusion criteria or about the disease state of the asthmatic population.

After *ex vivo* stimulation of heparinized whole blood with zymosan we observed significantly reduced levels of almost all main prostaglandins as well as of TXB_2_ in severe asthmatics. In contrast, metabolites of 5 and 15-LOX pathway LTE_4_ and 15HETE revealed increased concentrations in mild-to-moderate asthmatics but not in patients with severe disease compared to healthy controls. This finding was unexpected as previous studies showed a correlation between urinary and plasma LTE_4_ levels with asthma severity.^31^^,^^32^ In addition to administration of inhalative steroids with predominant local activities, severe asthmatics are often treated with oral corticosteroids which are known to inhibit expression of COX-2 isoform.^33^ Therefore, we analyzed our data for a potential effect of OCS therapy on metabolite levels and found a significant decrease in metabolite concentrations for all compounds of the COX pathway in severe asthmatics which use OCS on a daily basis. In contrast, leukotrienes and other metabolites of the different LOX pathways are less affected by OCS intake. Misso et al. and Aggarwal et al. observed reduced sputum and urinary LTE_4_ levels in severe asthmatics compared to mild-to-moderate subjects and suggested a potential effect of OCS intake for the severe group.^34^^,^^35^ The results presented in this study confirmed this hypothesis, as we observed a tendency towards decreased LTE_4_ levels in the severe asthma group using OCS compared to the OCS naïve patients, but this did not reach statistical significance. However, OCS naïve patients with severe asthma show an increased LTE_4_ synthesis compared to healthy subjects but were comparable to the mild-to moderate group. Although we could not exclude, that severe asthmatics may have shown even higher LTE_4_ levels before OCS treatment, our data suggest only a limited effect of oral corticosteroids in controlling the synthesis of cysteinyl leukotrienes, as the precursors of LTE_4_ were not affected. Therefore, additional treatment e.g., with anti-leukotriene drugs may help to improve symptom control in severe asthma.

Conflicting results are reported in the literature about the effect of oral glucocorticoids on the expression of prostaglandins. In a recent study, urinary PGD_2_ levels did not change in severe asthmatics dependent on OCS compared to OCS naïve asthmatics.^12^ In addition, short term use of prednisone had no effect on eicosanoid levels in the bronchoalveolar lavage of atopic asthmatics.^36^ On the other hand, prednisone was shown to suppress prostaglandin and TXB_2_ release in human polymorphonuclear leukocytes and in macrophage enriched BAL cells of healthy subjects.^37^^,^^38^ In this study, we found a strongly decreased expression of all main prostanoids in patients using OCS regularly, being in line with the reduced expression of COX2 observed in asthmatic patients on prednisolone therapy.^33^ It is suggested that inhibition of prostaglandin synthesis in asthma may indirectly amplify leukotriene biosynthesis, as arachidonic acid would be channeled towards other eicosanoid biosynthesis pathways,^4^ thus probably having an adverse effect on disease progression. However, our results do not support this hypothesis as OCS treated asthmatics did not show increased levels of leukotrienes or other LOX metabolites.

Despite the effect of OCS on eicosanoid production found in our study, a few metabolites were still differentially expressed in OCS naïve asthmatics. From these differentially expressed metabolites, effects of LTE_4_ on asthma pathophysiology have been extensively studied.^4^ In contrast, the role of 15-HETE in the development of allergic airway inflammation is not yet fully understood. Increased 15-HETE levels have been found in induced sputum from asthmatic patients compared to heathy controls.^39^ Furthermore, 15-HETE levels were elevated in the BAL fluid of severe eosinophilic asthmatics compared to non-eosinophilic airway inflammation.^40^ In a model of experimental asthma 15-LOX deficient mice were protected from allergen induced airway inflammation, suggesting a pro-inflammatory role for 15-HETE in asthma.^41^ In contrast, inhalation of 15-HETE did not directly influence lung function or induce bronchoconstriction in asthmatic patients or healthy controls and attenuates airway responsiveness towards histamine and methacholine.^42^ In our study we observed a distinct pattern of 15-HETE expression, as it is increased in mild-to-moderate asthmatics compared to severe asthmatics and healthy controls. Similar observations were made, when heparinized whole blood of asthmatic patients were stimulated with a calcium ionophore.^43^ The generation of 15-HETE and LXA_4_ was markedly decreased in severe asthmatics compared to the moderate group, as well as transcription of 15-LOX mRNA.^43^ 15-HETE serves as precursor for the synthesis of lipoxins (LXA_4_ and LXB_4_) which have shown to possess anti-inflammatory properties.^44^ Thus, decreased 15-HETE levels in severe asthmatics may be connected to a decreased potential to synthesize anti-inflammatory lipid mediators, contributing to the pathophysiology of severe asthma.

When analyzing eicosanoids from blood samples, the choice of an appropriate sampling system is mandatory in order to obtain reliable results, as eicosanoids can be formed artificially *ex vivo* by activated blood cells after venipuncture and through autooxidation processes in the collected blood. A massive increase in eicosanoid formation in serum due to platelet activation has been shown.^17^^,^^45^^,^^46^ That is why we used plasma instead of serum for our measurement. Furthermore, EDTA plasma is superior over heparin plasma, as especially 12-LOX metabolites, and TXB_2_ were found at higher concentrations in heparin plasma.^45^^,^^47^ This could be presumably attributed to heparin induced platelet activation^48^ or still activated enzymes. The latter is prevented by use of EDTA plasma due to the non-reversible chelation of Mg^2+^ and Ca^2+^ ions. Further strengths of our current study include the utilization of samples, derived by the well-characterized multicenter ALLIANCE cohort, which recruited patients according to the ERS/ATS guideline, as well as well-matched healthy controls providing robust comparability. Also, to the best of our knowledge, our study is the first to analyze whole blood-stimulated samples, thus providing data on the physiologically relevant blood cell output related to eicosanoid biosynthesis. We acknowledge that our study is also subject to limitations, since we cannot directly connect changes in eicosanoid concentrations to specific cell sources. This would require isolation of individual peripheral blood cell subsets, followed by ex-vivo stimulation in culture or co-culture systems. Moreover, we have not performed functional assays and did not compare our data with levels of mediators following incubations with other relevant stimuli such as LPS^49^ or β-Glucan.^50^ Finally, whole blood samples do not fully replicate the in vivo inflammatory environment, particularly in the lungs, thus limiting sensitivity and generalizability.

## Conclusions

After whole blood stimulation asthmatics showed an increased capacity for the synthesis of LTE_4_ compared to healthy controls, contributing to the well-known pro-inflammatory potential of this eicosanoid in allergic asthma. Additionally, patients with severe asthma have an impaired ability for synthesis of 15-HETE a precursor for the synthesis of anti-inflammatory mediators. Systemic glucocorticoid therapy was associated with variations in eicosanoid biosynthesis pathways, as only prostaglandin metabolism was strongly suppressed, whereas biosynthesis of leukotrienes and other lipoxygenase metabolites was less affected. Therefore, we conclude that there is a preference for the production of pro-inflammatory eicosanoids in patients with asthma which is only partially attenuated by oral glucocorticoids. Giving the emerging role of LTE4 und the cysteinyl leukotrienes receptors, future studies should focus on evaluating its expression in different asthma phenotypes and its potential as a therapeutic target.^51^

## Abbreviations section

2-HHT, 12-Hydroxyheptadecatrienoic acid; BAL, Bronchoalveolar Lavage; COX, Cyclooxygenase; CYP450, Cytochrome P450 Monooxygenase (auch CYP genannt); DZL, German Center for Lung Research (Deutsches Zentrum für Lungenforschung); EDTA, Ethylenediaminetetraacetic acid; ERS/ATS, European Respiratory Society/American Thoracic Society; FCS, Functional Class Scoring; HETE, Hydroxy-eicosatetraenoic acids; HPLC-MS2, High-Performance Liquid Chromatography-Tandem Mass Spectrometry; LCMS, Liquid Chromatography-Mass Spectrometry; Leukotrienes, LTE4, LTD4, LTB4; Lipoxins, LXA4, LXB4; LOD, Limit of Detection; LOX, Lipoxygenase; MO, Missouri (location abbreviation for St. Louis, USA); OCS, Oral Corticosteroids; Prostaglandins, PGE2, PGF2α, PGA2, PGD2, PGI2, PGE1; Q1/Q3, Quadrupole 1/Quadrupole 3 (used in mass spectrometry); RPMI, Roswell Park Memorial Institute (a type of medium); TLR2, Toll-Like Receptor 2; TXB2, Thromboxane B2.

## Author contributions

CS, TB, HW, CH, KFR, HR, FB, GH, BS, SW and WAN conceived the idea and designed the experiments. TB, MA, HW, FP and CH performed the experiments. PT analyzed the data. CS, PT and WAN wrote the manuscript with input from all authors. All authors have accepted responsibility for the entire content of this manuscript and approved its submission.

## Research funding

This research was funded by project grants from the German 10.13039/501100002347Federal Ministry of Education and Research (BMBF) as part of the DZL funding (82DZL005A2).

CS is supported by the 10.13039/100031239Universities Giessen and Marburg Lung Center (UGMLC), the 10.13039/501100010564German Center for Lung Research (DZL), 10.13039/501100009560University Hospital Giessen and Marburg (UKGM) research funding according to article 2, section 3 cooperation agreement, and the 10.13039/501100001659Deutsche Forschungsgemeinschaft (DFG, 10.13039/501100001659German Research Foundation)-SFB 1021 (Project Number 197785619), KFO 309 (Project Number 284237345), and SK 317/1-1 (Project Number 428518790) as well as by the 10.13039/501100008799Foundation for Pathobiochemistry and Molecular Diagnostics.

## Declaration of competing interest

For CS, Consultancy and research funding, Bencard Allergie and Thermo Fisher Scientific.

The other authors state no conflict of interest.
