# Supplementary material for: Differential expression of eicosanoid pathways after whole blood stimulation in asthma patients
Source: World Allergy Organ J. 2025 Apr 2;18(4):101047. doi: 10.1016/j.waojou.2025.101047 (PMC11999605; doi:10.1016/j.waojou.2025.101047)
Supplement: Multimedia component 1 [file mmc1.docx]

**Supplemental Material**

**Differential expression of eicosanoid pathways after**

**whole blood stimulation in asthma patients**

**Supplemental Table 1:** Composition and final sample concentration of deuterated internal standard mixture used for MS based analysis of eicosanoids.

| **Abbreviation** | **Full Name/Synonym** | **Final sample concentration**  **[ng/ml]** |
| --- | --- | --- |
| 11,12-EET-d_11_ | (±)11,12-epoxyeicosatrienoic acid-d_11_ | 2.94 |
| 12,13-diHOME-d_4_ | (±)12,13-dihydroxyoctadecenoic acid-d_4_ | 0.98 |
| 12-HETE-d_8_ | 12(S)-hydroxyeicosatetraenoic acid-d_8_ | 4.90 |
| 13-HODE-d_4_ | 13(S)-hydroxyoctadecadienoic acid-d_4_ | 0.98 |
| 14,15-EET-d_11_ | (±)14,15-epoxyeicosatrienoic acid-d_11_ | 0.98 |
| 15d-PGJ2-d_4_ | 15-deoxy-Δ^12,14^-Prostaglandin J_2_-d_4_ | 0.98 |
| 15-HETE-d_8_ | 15(S)-hydroxyeicosatetraenoic acid-d_8_ | 0.98 |
| 20-HETE-d_6_ | 20-hydroxyeicosatetraenoic acid-d_6_ | 2.94 |
| 5,6-EET-d_11_ | (±)5,6-epoxyeicosatrienoic acid-d_11_ | 4.90 |
| 5-HETE-d_8_ | 5(S)-hydroxyeicosatetraenoic acid-d_8_ | 0.98 |
| 5-iso-PGF_2α_-d_11_ | (±)5-iso-Prostaglandin F_2α_ VI-d_11_ | 0.98 |
| 5-oxo-ETE-d_7_ | 5-oxo-eicosatetraenoic acid-d_7_ | 2.45 |
| 6-keto-PGF_1α_-d_4_ | 6-keto-Prostaglandin F_1α_-d_4_ | 0.98 |
| 8,9-EET-d_11_ | (±)8,9-epoxyeicosatrienoic acid-d_11_ | 2.45 |
| 9,10-diHOME-d_4_ | (±)9,10-dihydroxyoctadecenoic acid-d_4_ | 0.98 |
| 9-HODE-d_4_ | 9(S)-hydroxyoctadecadienoic acid-d_4_ | 0.98 |
| AA-d_8_ | Arachidonic acid-d_8_ | 9.80 |
| DHA-d_5_ | Docosahexaenoic acid-d_5_ | 4.90 |
| dhk-PGD_2_-d_4_ | 13,14-dihydro-15-keto-Prostaglandin D_2_-d_4_ | 0.98 |
| dhk-PGE_2_-d_4_ | 13,14-dihydro-15-keto-Prostaglandin E_2_-d_4_ | 0.98 |
| dhk-PGF_2α_-d_4_ | 13,14-dihydro-15-keto-Prostaglandin F_2α_-d_4_ | 2.94 |
| EPA-d_5_ | Eicosapentaenoic acid-d_5_ | 4.90 |
| LTB_4_-d_4_ | Leukotriene B_4_-d_4_ | 0.98 |
| LTC_4_-d_5_ | Leukotriene C_4_-d_5_ | 0.98 |
| LTE_4_-d_5_ | Leukotriene E_4_-d_5_ | 0.98 |
| PGB_2_-d_4_ | Prostaglandin B_2_-d_4_ | 0.98 |
| PGD_2_-d_4_ | Prostaglandin D_2_-d_4_ | 0.98 |
| PGE_2_-d_4_ | Prostaglandin E_2_-d_4_ | 0.98 |
| PGF_2α_-d_4_ | Prostaglandin F_2α_-d_4_ | 0.98 |
| RvE_1_-d_4_ | Resolvin E_1_-d_4_ | 2.45 |
| TXB_2_-d_4_ | Thromboxane B_2_-d_4_ | 0.98 |

**Supplemental Table 2:** Overview of eicosanoids analyzed in this study.

| **Abbreviation** | **Full Name/Synonym** |
| --- | --- |
| 10,17-DiHoHE | 10(R),17(S)-dihydroxydocosahexaenoic acid (NPD_1_) |
| 10-HDoHE | (±)10-hydroxydocosahexaenoic acid |
| 11,12-DHET | (±)11,12-dihydroxyeicosatrienoic acid |
| 11,12-EET | (±)11,12-epoxyeicosatrienoic acid |
| 11-dh-TXB_2_ | 11-dehydro-thromboxane B_2_ |
| 11-HEPE | (±)11-hydroxyeicosapentaenoic acid |
| 11-HETE | (±)11-hydroxyeicosatetraenoic acid |
| 12,13-diHOME | (±)12,13-dihydroxyoctadecenoic acid |
| 12,13-EpOME | (±)12(13)-epoxyoctadecenoic acid |
| 12-HEPE | (±)12-hydroxyeicosapentaenoic acid |
| 12-HETE | (±)12-hydroxyeicosatetraenoic acid |
| 12-HHT | 12-hydroxyheptadecatrienoic acid |
| 12-oxo-ETE | 12-oxo-eicosatetraenoic acid |
| 13-HODE | (±)13-hydroxyoctadecadienoic acid |
| 14,15-DHET | (±)14,15-dihydroxyeicosatrienoic acid |
| 14,15-diHETE | (±)14,15-dihydroxyeicosatetraenoic acid |
| 14,15-LTC_4_ | 14,15-leukotriene C_4_ (Eoxin C_4_) |
| 14-HDoHE | (±)14-hydroxydocosahexaenoic acid |
| 15d-PGJ2 | 15-deoxy-Δ^12,14^-prostaglandin J_2_ |
| 15-HEPE | (±)15-hydroxyeicosapentaenoic acid |
| 15-HETE | (±)15-hydroxyeicosatetraenoic acid |
| 15-oxo-ETE | 15-oxo-eicosatetraenoic acid |
| 17-HDoHE | (±)17-hydroxydocosahexaenoic acid |
| 17-keto-DPA | 17-keto-docosapentaenoic acid |
| 18-HEPE | (±)18-hydroxyeicosapentaenoic acid |
| 18-HETE | (±)18-hydroxyeicosatetraenoic acid |
| 20-COOH-LTB_4_ | 20-carboxy-leukotriene B_4_ |
| 20-HETE | (±)20-hydroxyeicosatetraenoic acid |
| 20-OH-LTB_4_ | 20-hydroxy-leukotriene B_4_ |
| 4-HDoHE | (±)4-hydroxydocosahexaenoic acid |
| 5,15-diHETE | (±)5,15-dihydroxyeicosatetraenoic acid |
| 5-HEPE | (±)5-hydroxyeicosapentaenoic acid |
| 5-HETE | (±)5-hydroxyeicosatetraenoic acid |
| 5-iso-PGF_2α_ | (±)5-iso-prostaglandin F_2α_ VI |
| 5-oxo-ETE | 5-oxo-eicosatetraenoic acid |
| 6,15-dkdh-PGF1_a_ | 6,15-diketo-13,14-dihydro-PGF1_α_ |
| 6-keto-PGF_1α_ | 6-keto-prostaglandin F_1α_ |

**Supplemental Table 2:** Continued.

| **Abbreviation** | **Full Name/Synonym** |
| --- | --- |
| 7,17-DiHDPA | 7,17-dihydroxydocosapentaenoic acid |
| 7-HDoHE | (±)7-hydroxydocosahexaenoic acid |
| 8,15-diHETE | (±)8,15-dihydroxyeicosatetraenoic acid |
| 8-HEPE | (±)8-hydroxyeicosapentaenoic acid |
| 8-HETE | (±)8-hydroxyeicosatetraenoic acid |
| 9,10-diHOME | (±)9,10-dihydroxyoctadecenoic acid |
| 9-HEPE | (±)9-hydroxyeicosapentaenoic acid |
| 9-HETE | (±)9-hydroxyeicosatetraenoic acid |
| 9-HODE | (±)9-hydroxyoctadecadienoic acid |
| 9-HpODE | (±)9-hydroperoxyoctadecadienoic acid |
| LTB_4_ | Leukotriene B_4_ |
| LTC_4_ | Leukotriene C_4_ |
| LTD_4_ | Leukotriene D_4_ |
| LTE_4_ | Leukotriene E_4_ |
| LXA_4_ | Lipoxin A_4_ |
| MaR_1 | Maresin 1 |
| PGA_2_ | Prostaglandin A_2_ |
| PGD_2_ | Prostaglandin D_2_ |
| PGD_3_ | Prostaglandin D_3_ |
| PGE_2_ | Prostaglandin E_2_ |
| PGE_3_ | Prostaglandin E_3_ |
| PGF_1α_ | Prostaglandin F_1α_ |
| PGF_2α_ | Prostaglandin F_2α_ |
| PGF_3α_ | Prostaglandin F_3α_ |
| RvD_2_ | Resolvin D_2_ |
| RvE_1_ | Resolvin E_1_ |
| tn-12-HETE | tetranor-12-HETE |
| tn-PGEM | tetranor-PGEM |
| TXB_2_ | Thromboxane B_2_ |
| TXB_3_ | Thromboxane B_3_ |

**
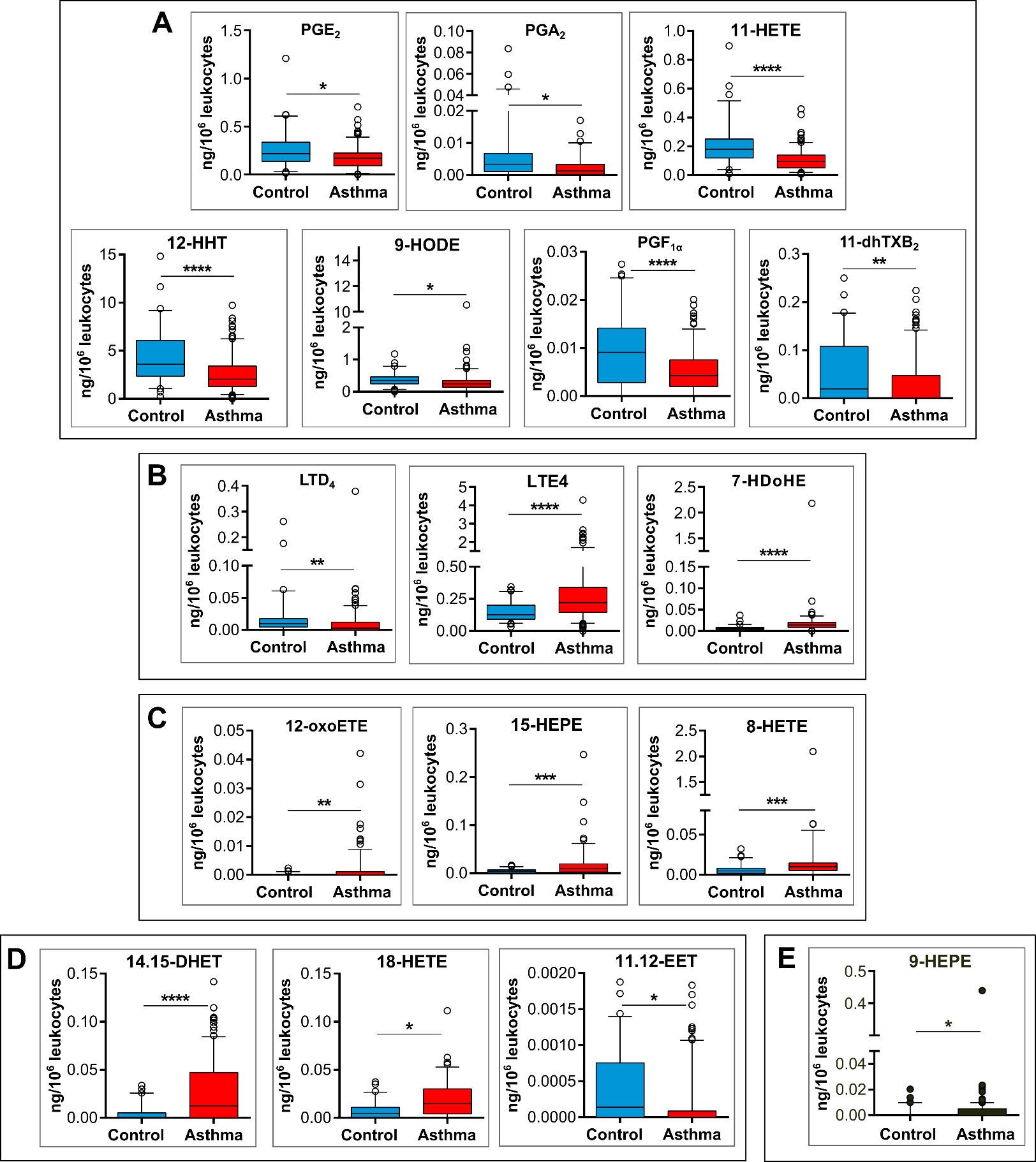
**

**Supplemental Figure 1:** Significantly regulated eicosanoids in asthmatic patients compared to healthy controls after whole blood stimulation with zymosan for 4h. A: COX metabolites, B: 5-LOX metabolites, C: 12/15-LOX metabolites, D: Cyp450 metabolites, E: metabolites formed by autooxidation. *p‑value ≤ 0.05, ** p-value ≤ 0.01, ***p-value ≤ 0.001, ****p-value ≤ 0.0001.


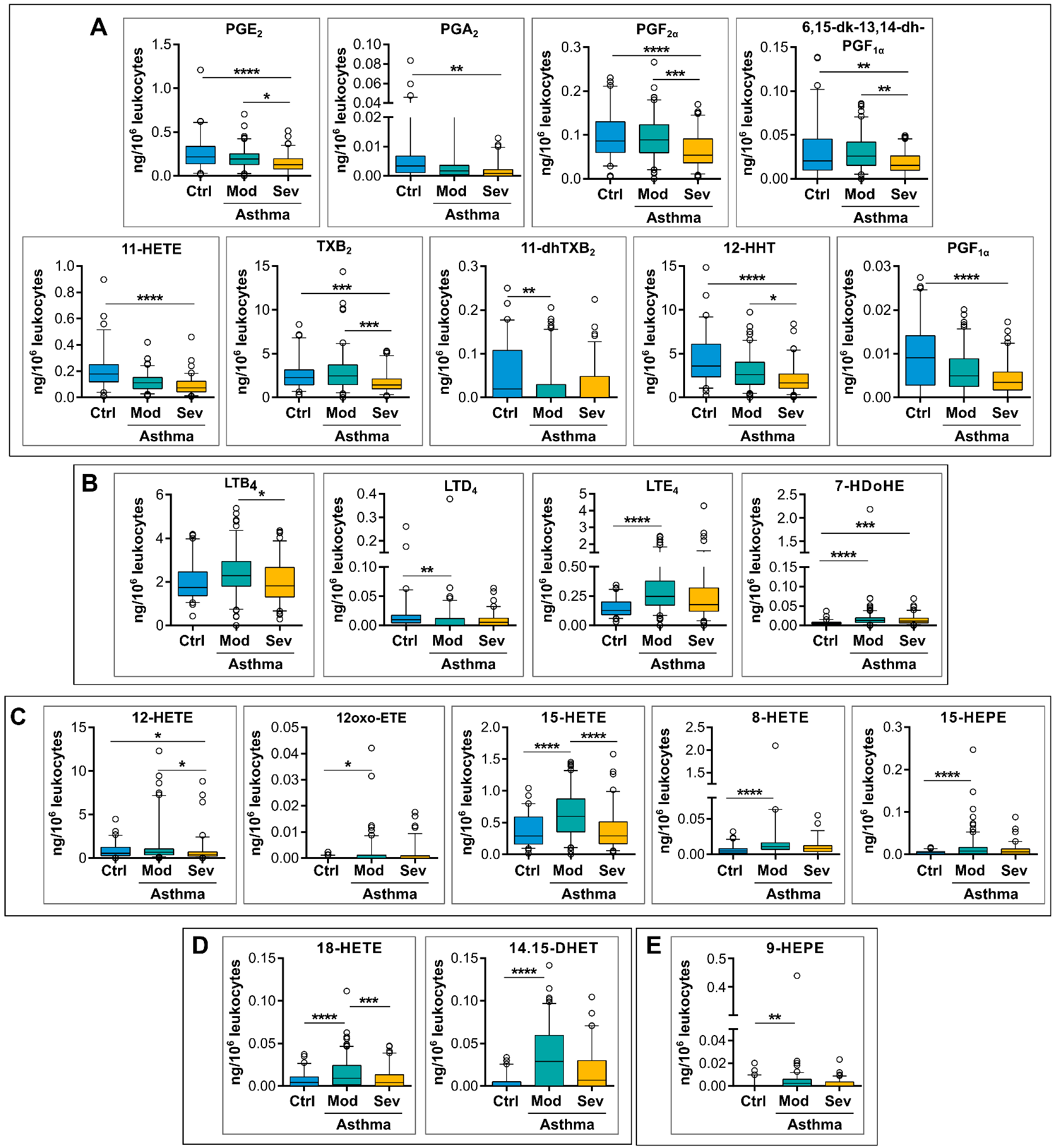


**Supplemental Figure 2:** Significantly regulated eicosanoids in mild-to-moderate (Mod) and severe (Sev) asthmatic patients compared to healthy controls (Ctrl) after whole blood stimulation with zymosan for 4h. A: COX metabolites, B: 5-LOX metabolites, C: 12/15-LOX metabolites, D: Cyp450 metabolites, E: metabolites formed by autooxidation. *p-value ≤0.05, ** p-value ≤ 0.01, ***p‑value ≤0.001, ****p‑value ≤0.0001.

**Supplemental References:**

SR1. Chung KF, Wenzel SE, Brozek JL, Bush A, Castro M, Sterk PJ, et al. International ERS/ATS guidelines on definition, evaluation and treatment of severe asthma. Eur Respir J 2014;43:343–73.

SR2. Dumlao DS, Buczynski MW, Norris PC, Harkewicz R, Dennis EA. High-throughput lipidomic analysis of fatty acid derived eicosanoids and N-acylethanolamines. Biochim Biophys Acta BBA - Mol Cell Biol Lipids 2011;1811:724–36

SR3. Banhos Danneskiold-Samsøe N, Sonne SB, Larsen JM, Hansen AN, Fjære E, Isidor MS, et al. Overexpression of cyclooxygenase-2 in adipocytes reduces fat accumulation in inguinal white adipose tissue and hepatic steatosis in high-fat fed mice. Sci Rep 2019;9:8979

SR4. R Core Team. R: A language and environment for statistical computing. Vienna, Austria: Foundation for Statistical Computing; 2020.

SR5. Kaplan EL, Meier P. Nonparametric Estimation from Incomplete Observations. J Am Stat Assoc 1958;53:457–81.

SR6. Tobin J. Estimation of Relationships for Limited Dependent Variables. Econometrica 1958;26:24.

SR7. Lee ET, Wang JW. Statistical methods for survival data analysis. 3rd ed. New York: J. Wiley; 2003.

SR8. Wilcoxon F. Individual Comparisons by Ranking Methods. Biom Bull 1945;1:80.

SR9. Westfall PH, Young SS. Resampling-based multiple testing: examples and methods for P-value adjustment. New York: Wiley; 1993.

SR10. Khatri P, Sirota M, Butte AJ. Ten Years of Pathway Analysis: Current Approaches and Outstanding Challenges. PLoS Comput Biol 2012;8:e1002375.

SR11. Pesarin F. Multivariate permutation tests: with applications in biostatistics. Chichester ; New York: J. Wiley; 2001.

SR12 Smith DL, Willis AL. A suggested shorthand nomenclature for the eicosanoids. Lipids 1987;22:983–6.

SR13. Samuelsson B, Hammarström S. Nomenclature for leukotrienes. Prostaglandins 1980;19:645–8.
